# Diffuse Coevolution between Two *Epicephala* Species (Gracillariidae) and Two *Breynia* Species (Phyllanthaceae)

**DOI:** 10.1371/journal.pone.0041657

**Published:** 2012-07-27

**Authors:** Jing Zhang, Shuxia Wang, Houhun Li, Bingbing Hu, Xiaofei Yang, Zhibo Wang

**Affiliations:** College of Life Sciences, Nankai University, Tianjin, China; University of Oxford, United Kingdom

## Abstract

The diffuse coevolution between two moth species (*Epicephala lativalvaris* and *E. mirivalvata*) and two plant species (*Breynia fruticosa* and *B. rostrata*) is reported based on field observations and indoor experiments conducted in Hainan and Fujian, China. Study results showed that the two *Epicephala* species jointly pollinated the two *Breynia* species, which led to a unique obligate pollination mutualism of two−to−two species specificity. A single *Epicephala* larva exclusively fed on seeds of host plants and developed to maturity by consuming all six seeds of each fruit, whereas a fraction of intact fruits were left to ensure the reproduction of plants within the whole population. Larvae of the two *Epicephala* species are competitive for resources; the population of *E. mirivalvata* is much smaller than that of *E. lativalvaris*, which has resulted from the differences in the female ovipositor structures and oviposition mode. The life history of *Epicephala* species highly coincides with the phenology of *Breynia* plants, and different phenology of *B. fruticosa* resulted in the different life history of the two *Epicephala* species in Hainan and Fujian. The natural hybridization of two host plants, possibly induced by the alternate pollination of two *Epicephala* species, is briefly discussed.

## Introduction

The obligate pollination relationship between plants and their seed-parasitic pollinators is perhaps one of the most specialized cases in mutualism between insects and plants [1]–[4]. Ehrlich and Raven defined coevolution as the interaction between two major groups of organisms with a close and evident ecological relationship, such as plants and herbivores [5]. In the known classical obligate pollination mutualism between figs–fig wasps and yuccas–yucca moths, figs and yuccas depend exclusively on pollination of fig wasps and yucca moths respectively, while sacrificing some seeds for larvae of fig wasps and yucca moths to feed [6], [7]. The obligate pollination mutualism between *Epicephala* moths of Gracillariidae and *Glochidion* plants of Euphorbiaceae was discovered in 2003 [3]. Three pollination relationships were proposed [8]: (1) one *Epicephala* species obligately pollinates one *Glochidion* species, (2) two *Epicephala* species jointly pollinate one *Glochidion* species, and (3) two *Epicephala* species jointly pollinate two closely related parapatric *Glochidion* species. The most distinctive floral features associated with the pollination mode are the structures of the pistils and stamens: styles are usually reduced to entire tips and medially fused, or filaments and anthers are variously fused [9]–[10].

An obligate pollination relationship between *Breynia vitis*-*idaea* and an unnamed *Epicephala* species was discovered in 2004 [11]. This *Epicephala* species actively pollinated *B. vitis*-*idaea* and was observed loading numerous pollen grains on proboscis. Another *Epicephala* species on leaves of *B. fruticosa* was also observed carrying numerous pollen grains on proboscis, which led to a deduction that this species was an active pollinator of *B. fruticosa* [11]. Okamoto et al. [12] suggested the floral scent of *Glochidion* is one of the important key signals that mediate the encounters of the species-specific partners in the *Glochidion*–*Epicephala* mutualism. In tightly coevolved mutualism between *Breynia vitis-idaea* and *Epicephala* species, system-specific chemistry is not necessary for efficient host location to *Epicephala* moths [13].

By means of field observations and indoor experiments, we studied the phenology, the phylogenetic relationship and the morphological characters of the two *Breynia* species, as well as the morphological characters, the behavior, the life history, the phylogenetic relationship and the population size of the two *Epicephala* species in Hainan and Fujian. Based on related literature and results of our study, we discussed herein: (1) the impact of the different phenology of the allopatric *B. fruticosa* populations on the life history of the *Epicephala* moths in coevolution; (2) the influence of moth pollinators on the hybridization of *Breynia* species; and (3) whether the relationship between the pollinators is stable in this diffuse coevolution.

**Figure 1:**
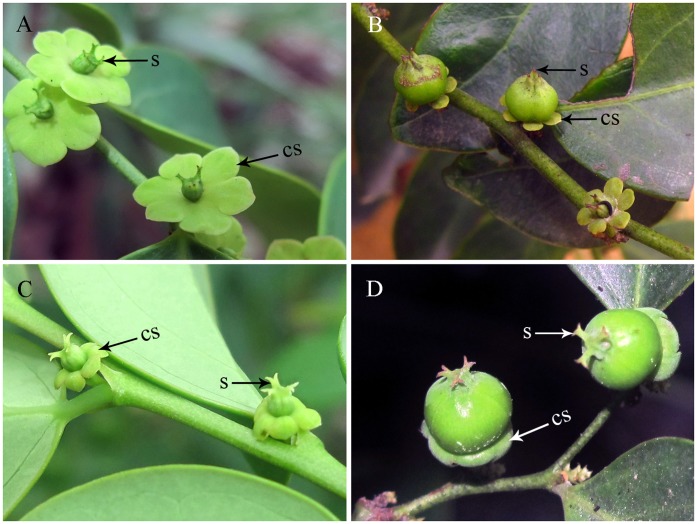
Female flowers and fruits with double characters of *Breynia* plants. Female flowers (A) and fruits (B) with erect stigmas and flat calyx sepals. Female flowers (C) and fruits (D) with excurved stigmas and reflexed calyx sepals. (cs) calyx sepals. (s) stigma.

## Results

### Life History of *Epicephala* Moths and Phenology of *Breynia* Plants

In Hainan, *B. fruticosa* has six fruit periods per year, lasting from the first period in early January to the sixth period in late November. *Epicephala lativalvaris* and *E. mirivalvata* have six generations correspondingly, lasting from the first generation in middle January to the sixth generation in late December (Table S1).

In Fujian, both *B. fruticosa* and *B. rostrata* have 4–5 fruit periods per year, which lasted from the first period in early June to the fifth period in late April of the following year. A large number of flowers withered in winter due to absence of pollinators. A small amount of flowers that were pollinated during middle to late November were dormant and no seeds were formed until middle or late March of the following year, and the fifth fruit period lasted from middle to late April. Both *E. lativalvaris* and *E. mirivalvata* have 4–5 generations correspondingly, which lasted from the first generation in middle June to the fifth generation in middle May of the following year. Larvae that pupated during early to middle November could emerge into adults, pollinate mature female flowers and lay eggs of the fifth generation during middle to late November. Larvae that pupated in late November overwintered in pupas, and they began to emerge in late April and disappeared in middle May. Pollinated flowers and *Epicephala* eggs in flowers were dormant until middle or late March of the following year, and the adults of the fifth generation emerged in middle May (Table S2). We smelled flower fragrance of both *B. fruticosa* and *B. rostrata* at night only, which suggests that they were blooming at night.

A single larva of both *E. lativalvaris* and *E. mirivalvata* needs to consume all six seeds within a fruit to develop into maturity. Mature larvae (the last instar) gnawed a hole in the fruit wall and exited, then produced cocoons and turned into pupae on leaves of hosts or the ambient plants. Pupal stage of *E. lativalvaris* lasted 9–15 days, and pupal stage of *E. mirivalvata* lasted 9–12 days. Adults of both *Epicephala* species could survive 3–5 days by sucking nectar for nutrition (Figure S1).

**Figure 2:**
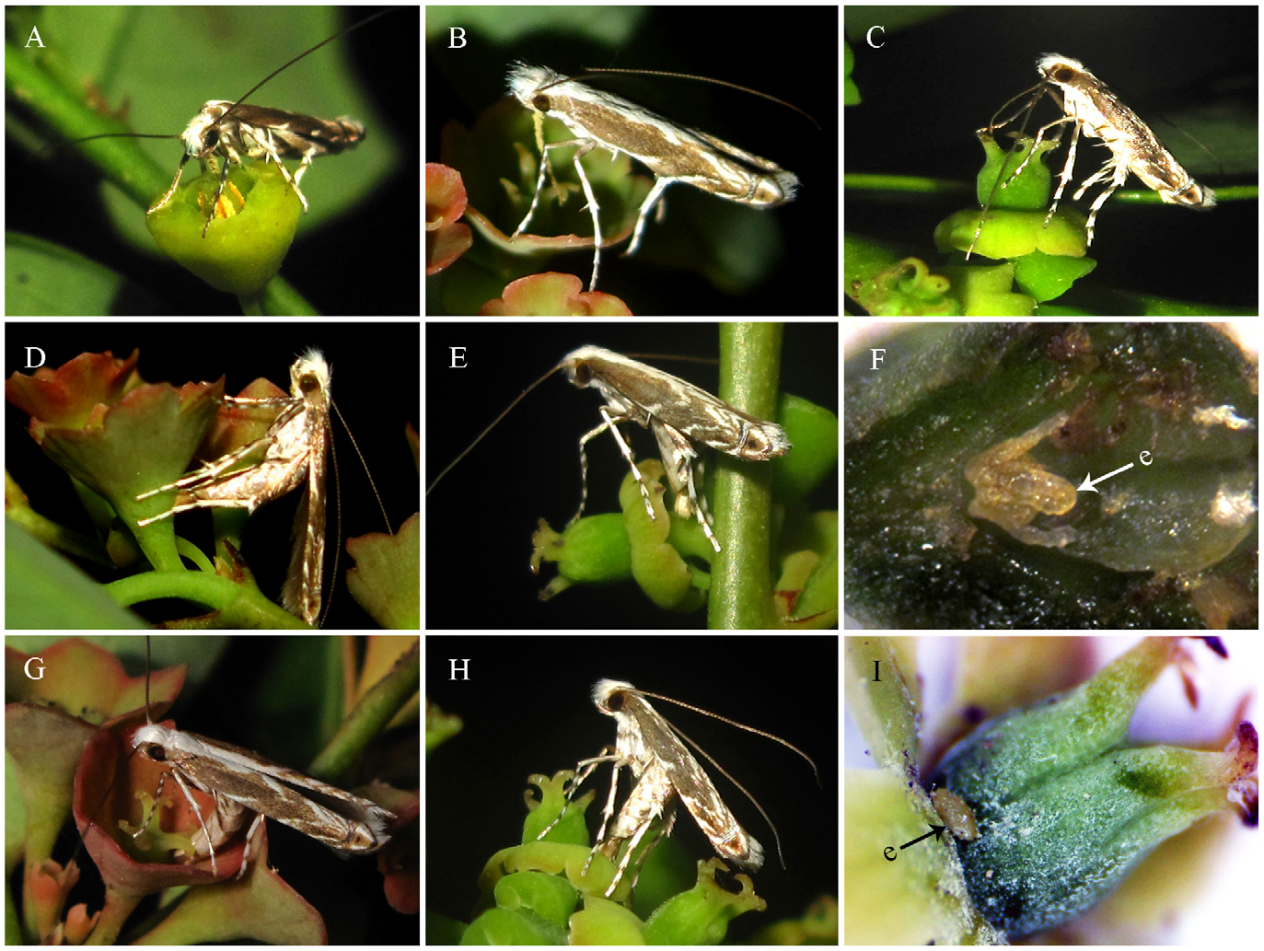
Behavior of female *Epicephala* moths. *Epicephala lativalvaris* collecting pollen grains on male flower of *Breynia fruticosa* (A) and actively pollinating for *Breynia fruticosa* (B) and *B. rostrata* (C). *E. lativalvaris* inserting ovipositor through calyx lobe and ovary to lay eggs on *B. fruticosa* (D) and *B. rostrata* (E). (F) Egg of *E. lativalvaris*. *E. mirivalvata* laying eggs between ovary and calyx lobe on *B. fruticosa* (G) and *B. rostrata* (H). (I) Egg of *E. mirivalvata*. (e) egg.

### Hybridization of *Breynia* Species

We found some plant individuals are difficult to identify, because they possess double morphological characters of both *B. fruticosa* and *B. rostrata*: three individuals (Figure 1: A, B) in Hainan with erect stigmas (characteristic of *B. rostrata*) and flat calyx sepals (characteristic of *B. fruticosa*); nineteen individuals (Figure 1: C, D) in Fujian with excurved stigmas (characteristic of *B. fruticosa*) and reflexed calyx sepals (characteristic of *B. rostrata*).

The result of the hand-pollination hybridization experiment showed that there are no reproductive barriers between *B. fruticosa* and *B. rostrata*. 90% *B. fruticosa* female flowers (n = 50) with pollen grains from *B. rostrata* developed, so did 94% *B. rostrata* female flowers (n = 50) with pollen grains from *B. fruticosa* (Table S7).

### Behavior of *Epicephala* Adults

*Epicephala* moths became active after dark, which was in accordance with the blossom of *B. fruticosa* and *B. rostrata* flowers. Female adults of the two *Epicephala* species actively collected pollen from male flowers and pollinated female flowers of both *Breynia* species at night. Female *Epicephala* adults pushed proboscis tip into the dehiscent pit of calyx lobes, rubbed against stamen column to collect pollen grains, and sometimes they pushed the whole proboscis into the pit (Figure 2: A). During pollen collection, female adults intermittently spread proboscis several times to get more pollen grains. They only collected pollen grains in one male flower each time effectively, which could pollinate more than ten female flowers in the same or different plants.

*Epicephala* adults repeatedly rubbed proboscis against stigma to deposit pollen grains on all stigmas (Figure 2: B, C). They would pace back and forth to pollinate all flowers on one branch, and female flowers were pollinated in this way as to pollinate most efficiently.

The two *Epicephala* species exhibited similar behavior of pollen collection and pollination, but their oviposition behavior was visibly different. *Epicephala lativalvaris* females bent abdomen to basal calyx on ventral surface and inserted ovipositor through calyx lobes and ovary to lay eggs into ovary tissue (Figure 2: D–F). *Epicephala mirivalvata* females bent abdomen to basal calyx on dorsal surface to deposit eggs between ovary and calyx lobes (Figure 2: G–I). After oviposition, *Epicephala* moths often gave complementary pollination to make sure there are enough pollen grains for female flowers. On rare occasion, they would not lay eggs if disturbed.

**Figure 3:**
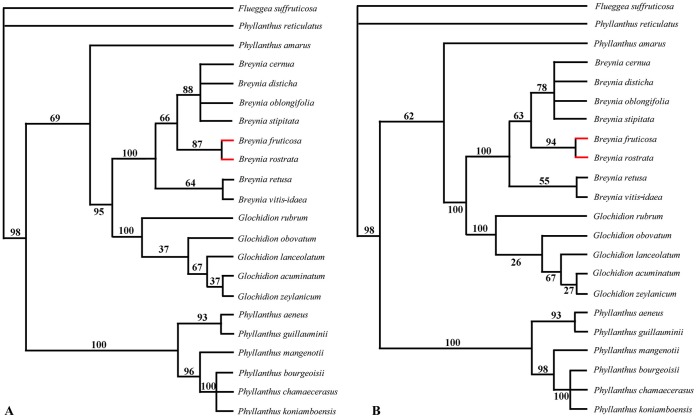
Maximum-parsimonious (left) and maximum-likelihood (right) trees based on *matK* genes of 22 Phyllantheae **plant species.** (A) The maximum-parsimonious tree of Phyllantheae plants (length, 179; CI = 0.933; RI = 0.985). (B) The maximum-likelihood tree of Phyllantheae plants (-ln likelihood = 2922.8917; transition/transversion ratio  = 2; empirical frequencies: A = 0.30688, C = 0.16491, G = 0.15206, T = 0.37615). Numbers above branches are bootstrap values. Red branches refer to the species involved in this study.

### Number of *Epicephala* Eggs and Consumption of *Breynia* Seeds

*Epicephala lativalvaris* laid eggs by piercing through calyx lobes and ovary wall, leaving oviposition scars on fruits and calyx lobes. We could acquire their egg amount by counting scars on each fruit. In all sampled fruits (n = 1269 in *B. fruticosa*, n = 867 in *B. rostrata*), 72.0% in *B. fruticosa* (n = 914) and 88.6% in *B. rostrata* (n = 768) with oviposition scars loaded *E. lativalvaris* eggs (Table S3). *Epicephala mirivalvata* laid eggs between calyx lobes and ovary wall. Therefore their eggs could only be observed by dissecting female flowers. Only 2.6% female flowers in *B. fruticosa* (n = 2) and 4.0% in *B. rostrata* (n = 5) loaded *E. mirivalvata* eggs in all dissected flowers (n = 76 in *B. fruticosa*, n = 126 in *B. rostrata*) (Table S4). We collected 28 *E. lativalvaris* adults and two *E. mirivalvata* adults in the field, and reared 109 *E. lativalvaris* larvae and four *E. mirivalvata* larvae from fruits. We observed *E. lativalvaris* visiting female flowers 35 times in the field, but *E. mirivalvata* twice only. The above statistical figures indicated that the population size of *E. mirivalvata* was much smaller than that of *E. lativalvaris*.

### Phylogenetic Analysis of *Epicephala* Moths and *Breynia* Plants

We analyzed the *COI* genes of *E. lativalvaris* and *Epicephala* sp. ex *B. fruticosa* [11], and found that the pairwise distance between them is 0. Moreover, *Epicephala* sp. ex *B. fruticosa* would leave scars on host flowers after ovipositing, which is similar to *E. lativalvaris*. Based on the above information, we could conclude that the *Epicephala* species pollinating *B. fruticosa* in Kawakita and Kato [11] is *E. lativalvaris*.

**Figure 4:**
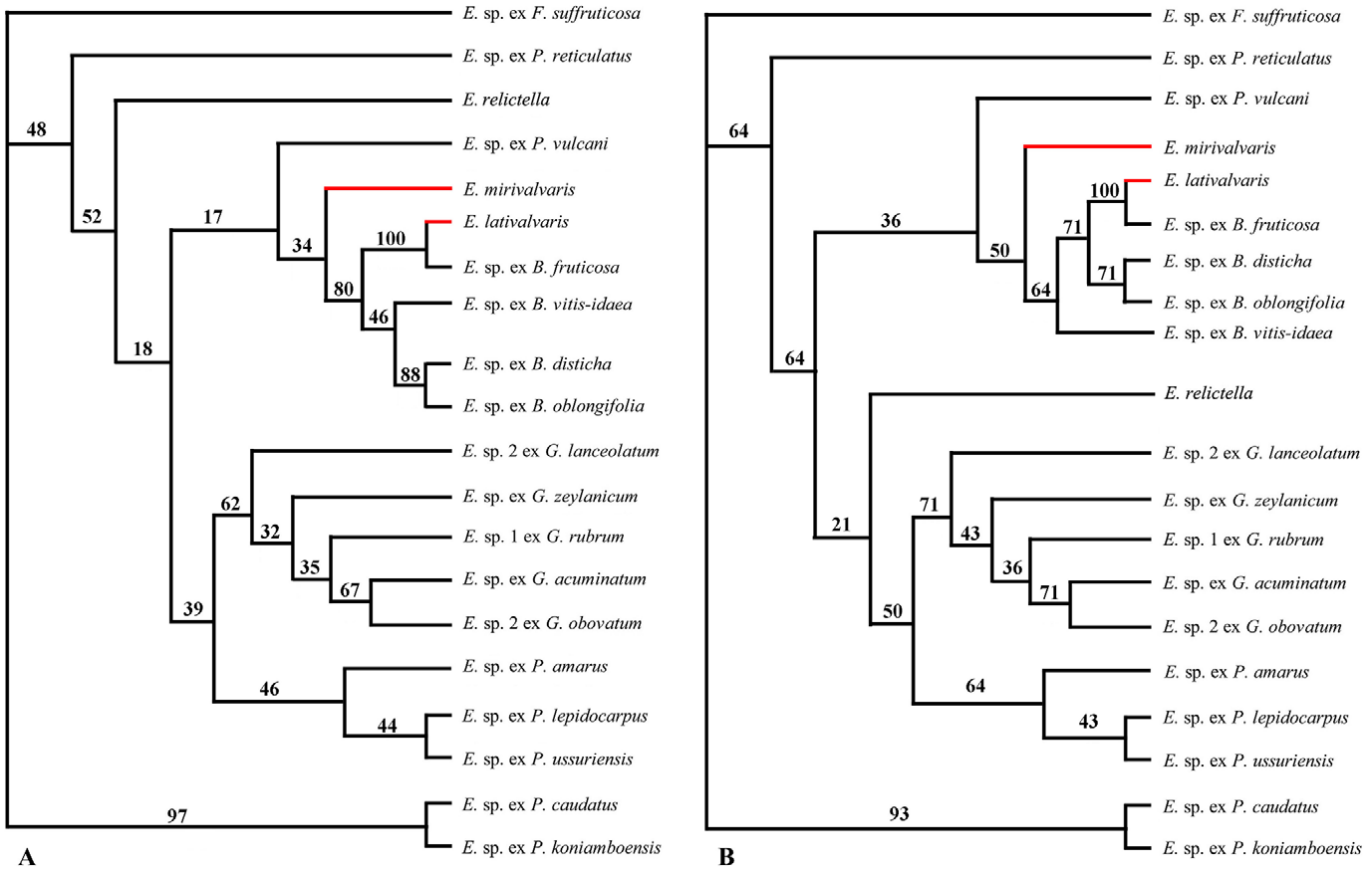
Maximum-parsimonious (left) and maximum-likelihood (right) trees based on *COI* genes of 19 *Epicephala* moths species. (A) The maximum-parsimonious tree of *Epicephala* moths (length, 486; CI = 0.519; RI = 0.518). (B) The maximum-likelihood tree of *Epicephala* moths (-ln likelihood = 1628.20777; transition/transversion ratio  = 2; empirical frequencies: A = 0.29923, C = 0.16930, G = 0.14101, T = 0.39046). Numbers above branches are bootstrap values. Red branches refer to the species involved in this study.

The topological structure of the maximum-parsimonious (MP) tree and the maximum-likelihood (ML) tree of plants are completely consistent (Figure 3), which indicates that *B. fruticosa* and *B. rostrata* are highly surpported to be sister taxa. Both MP and ML trees of *Epicephala* moths (Figure 4) showed that within five *Epicephala* pollinators of *Breynia* plants, *E. mirivalvata* diverged firstly from *E. lativalvaris*, *Epicephala* sp. ex *B. vitis-idaea*, *Epicephala* sp. ex *B. disticha* and *Epicephala* sp. ex *B. oblongifolia*, and that all of pollinators of *Breynia* have a close relationship with *Epicephala* sp. ex *P. vulcani*. The pollinators of *Glochidion* plants and three non-pollinators of *Phyllanthus* form a paraphyletic group.

## Discussion

### Diffuse Coevolution between Two *Epicephala* Species and Two *Breynia* Species

Diffuse coevolution firstly advanced and defined by Janzen (1980) [14]. He defined coevolution as an evolutionary change in a trait of the individuals in one population in response to a trait of the individuals of a second population, followed by an evolutionary response by the second population to the change in the first. Diffuse coevolution occurs when either or both populations in coevolution are represented by an array of populations that generate a selective pressure as a group [14].

**Figure 5:**
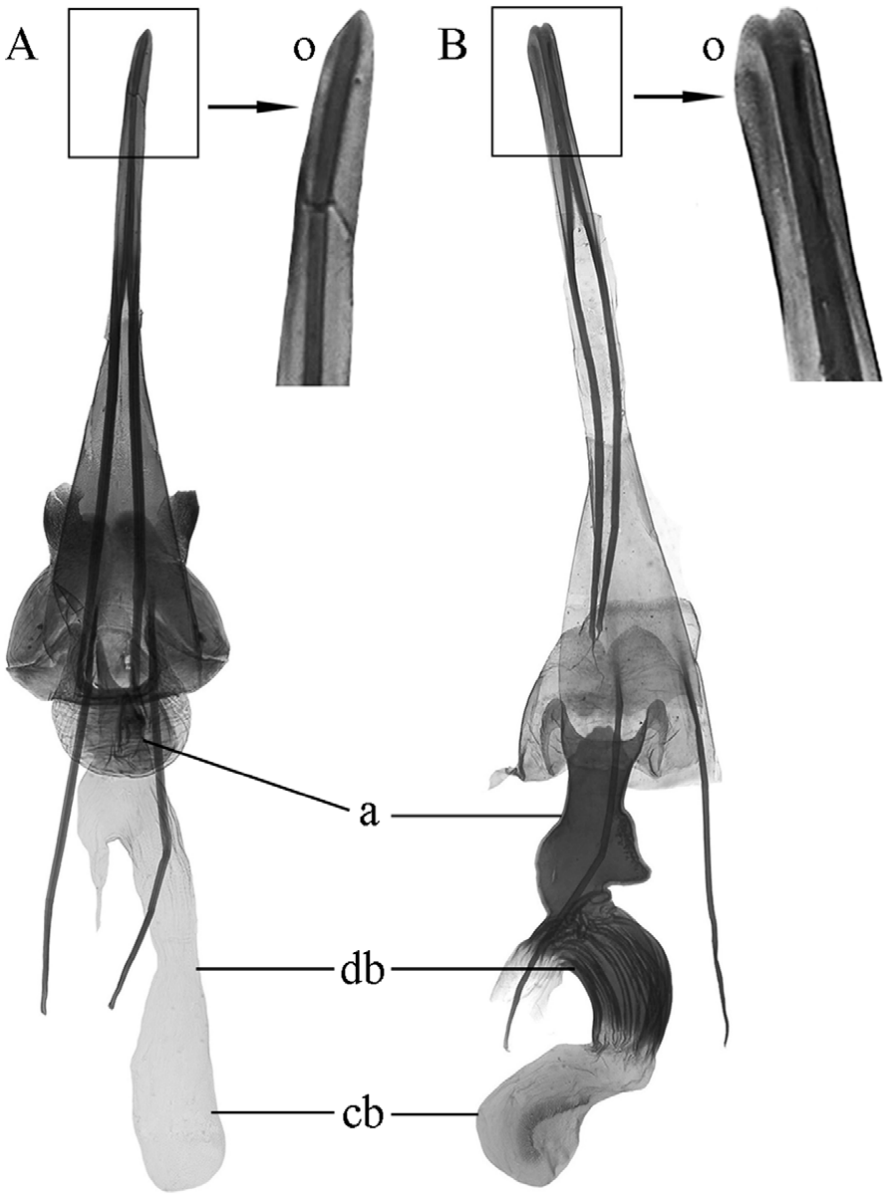
Morphological characters of *Epicephala* female genitalia. (A) *E. lativalvaris* with spine-shaped apex. (B) *E. mirivalvata* with blunt apex. (o) ovipositor. (a) antrum. (db) ductus bursae. (cb) corpus bursae.

Our study revealed that *B. fruticosa* and *B. rostrata* distributed in Hainan and Fujian were dependent on the obligate pollination of *E. lativalvaris* and *E. mirivalvata*, forming a mutualism of two−to−two species specificity. Two *Epicephala* species deposited eggs on female flowers before or after pollination, and their larvae exclusively fed on seeds of two *Breynia* species. The life history of two *Epicephala* species correlated with the phenology of two *Breynia* species; *Breynia* plants bloomed after dark, which coincided with the activity of *Epicephala* moths. According to the definition of diffuse coevolution and the classic examples of figs–fig wasps and yuccas–yucca moths, we confirm that diffuse coevolution exists between the two *Epicephala* species and the two *Breynia* species, which is firstly revealed in this study. Though a single *Epicephala* larva needs to consume all six seeds of each fruit to develop to maturity, enough intact fruits developed by *Epicephala* pollinated flowers are left to ensure the reproduction of the hosts within the whole population.

**Figure 6:**
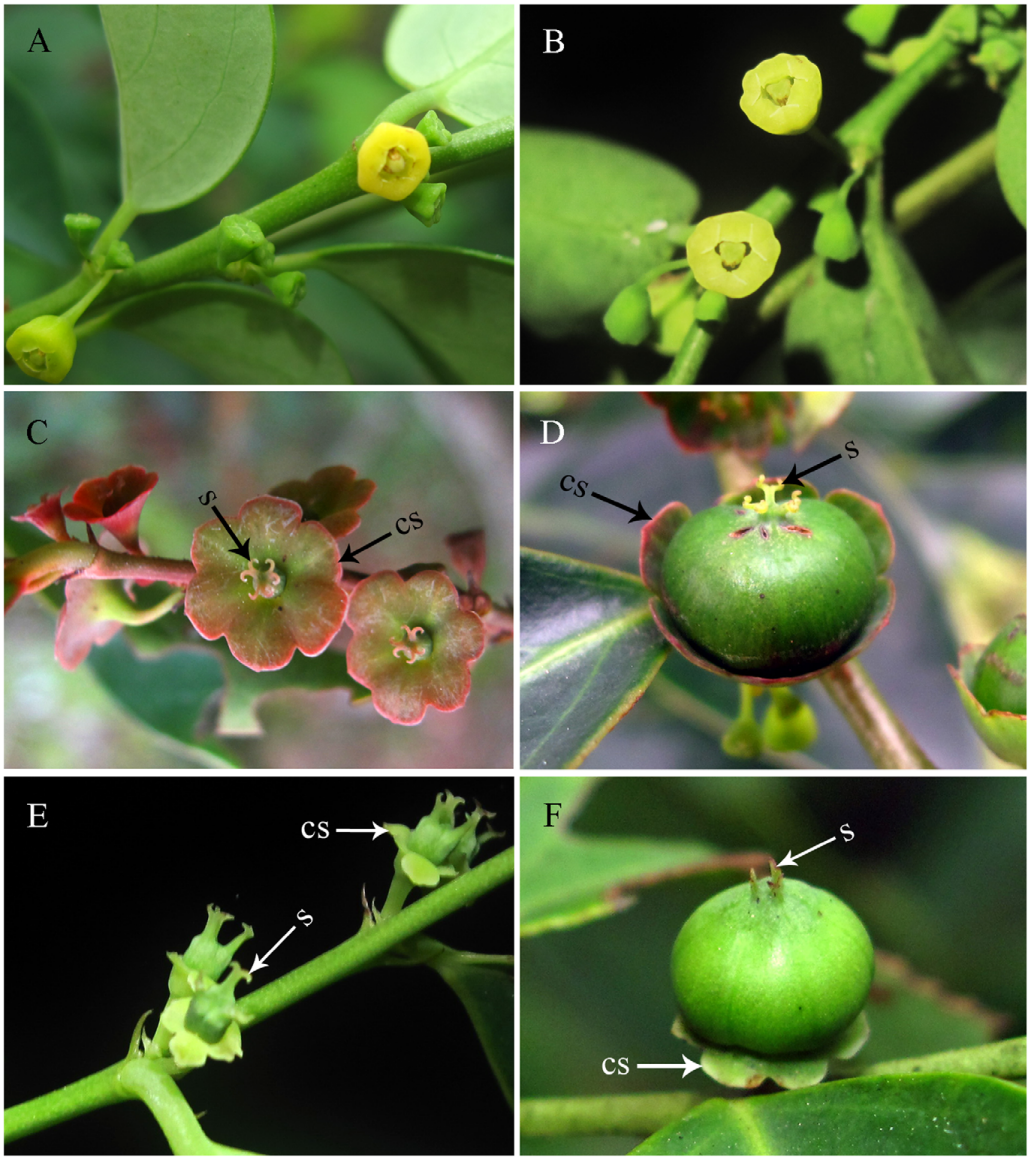
Flowers and fruits of *Breynia* plants. Male flowers of *B. fruticosa* (A) and *B. rostrata* (B) with stamens concealed in calyxe which can be visited by *Epicephala* moths only. Female flowers (C) and fruit (D) of *B. fruticosa* with excurved stigmas and discal calyx sepals. Female flowers (E) and fruit (F) of *B. rostrata* with stigmas erect and reflexed calyx sepals. (cs) calyx sepals. (s) stigma.

The impact of the phenology of the allopatric *B. fruticosa* on the life history of *Epicephala* moths was obvious. Different climates in Hainan and Fujian led to different phenology of *B. fruticosa*, which resulted in different life history of *Epicephala* species. In Hainan *B. fruticosa* has six fruit periods without dormant stage, so do two *Epicephala* species (Table S1). While in Fujian, pollinated female flowers of *B. fruticosa* were dormant in winter, so a minority of *Epicephala* individuals overwintered in eggs in female flowers and a majority overwintered in pupas until hosts bloom in the following year (Table S2).

*Breynia fruticosa* and *B. rostrata* are sister taxa, which suggests that the pollinators they are sharing might have pollinated their ancestor, and that this pollination system maintained after the speciation of *B. fruticosa* and *B. rostrata*.

### Hybridization of Two *Breynia* Plants Induced by *Epicephala* Moths

The study of the genetic structure of closely related figs and their associated pollinators in the Neotropical Region showed that shared use of host figs and colonization of novel hosts by the wasps may result in hybridization and genetic introgression across different fig species [15]. We found similar phenomena in *Breynia* plants. Among the four species of *Breynia* plants in China, *B. fruticosa* and *B. rostrata* are closely related in morphology. Stamens concealed in closing calyx distinguish them from *B. retusa*, and free calyx and styles distinguish them from *B. vitis-idaea*. The phylogenetic analysis based on *matK* gene also revealed that *B. fruticosa* and *B. rostrata* are sister taxa (Figure 3). *Breynia fruticosa* and *B. rostrata* distributed in Wanshi Botanical Garden and Tianzhu Mountain have similar phenology. When *Epicephala* moths collected pollen grains from male flowers of *B. fruticosa*, they are likely to deposit pollen grains on female flowers of the sympatric *B. rostrata*, hence resulting in the hybridization of *B. fruticosa* and *B. rostrata*, and vice versa. As *B. fruticosa* and *B. rostrata* are closely related, their hybrid offsprings could survive and possess double characters of both species. This hypothesis is supported by the fact that some plant individuals in Tianzhu Mountain and Wanshi Botanical Garden have double characters of both *B. fruticosa* and *B. rostrata* (Figure 1). *Breynia rostrata* was recorded to have distribution in Hainan [16], but we did not find it in this study. We only found three individuals with characters of both *B. rostrata* and *B. fruticosa* in Yingge Mountain, which indicated that the population of *B. rostrata* was either very small or has disappeared. The plants with double characters in Hainan may imply that *B. rostrata* once existed there and might have hybridized with *B. fruticosa* in nature.

### Relationship between *E. Lativalvaris* and *E. Mirivalvata*

In *Breynia*–*Epicephala* diffuse coevolution mutualism, *Epicephala lativalvaris* and *E. mirivalvata* overlap in the ecological niche, and are competitive for resources. *Epicephala mirivalvata* has a much smaller population size than *E. lativalvaris* does and plays a secondary role in this mutualism. This is closely associated with female ovipositor structures and oviposition mode. *Epicephala lativalvaris* has an apically spine-shaped ovipositor (Figure 5: A), which could pierce through calyx lobes and ovary wall to lay eggs into the ovary tissue (Figure 2: D–F). So the external influence imposed on egg hatching could be greatly reduced, and the newly hatched larvae could instantly feed on seeds to ensure a higher survival rate. On the contrary, *Epicephala mirivalvata* has a clavate ovipositor with blunt apex (Figure 5: B), which could deposit eggs only between calyx lobes and ovary (Figure 2: G–I). So both eggs and newly hatched larvae were exposed to the influence of environmental factors. Moreover, the newly hatched larvae had to bore into the ovary, which could increase the mortal hazard and eventually decrease the population size. The phylogenetic analysis of *Epicephala* trees reveals that *E. mirivalvata* diverged firstly within all known pollinators of *Breynia* plants, that *E. mirivalvata* firstly established relationship with *B. fruticosa* and *B. rostrata*. However, its ovipositior structure and oviposition mode have resulted its inferiority in competition with *E. lativalvaris*. So it can be deduced that *E. mirivalvata* is likely to disappear or shift host to maintain population size, and *E. lativalvaris* may become the sole pollinator of *B. fruticosa* and *B. rostrata* in the future evolutionary course.

### Conclusion

In *Breynia*–*Epicephala* diffuse coevolution, two morphologically and behaviorally distinct *Epicephala* species jointly pollinated two *Breynia* species, forming a new obligate pollination mutualism of two–to–two species speciality. We firstly describe this model of mutualism system in this study. Different phenology of *B. fruticosa* resulted in different life history of *Epicephala* species in Hainan and Fujian, and two hosts sharing pollination of the two *Epicephala* species has led to hybridization of *Breynia* plants. In this mutualism, two competitive *Epicephala* species overlap in the ecological niche. *Epicephala mirivalvata* is much smaller than *E. lativalvaris* in population size due to different oviposition mode, and therefore faces more selection pressure.

## Materials and Methods

### Study Sites

From November 2009 to September 2011, we carried out field observations on *Breynia fruticosa* in Yingge Mountain Nature Reserves (alt. 200–755 m, 18°50′–19°12′N, 109°15′–109°50′E), Wuzhi Mountain National Nature Reserves (alt. 512–720 m, 18°49′–18°59′N, 109°32′–109°43′E) and Jianfeng Mountain National Nature Reserves (alt. 807–973 m, 18°23′–18°52′N, 108°44′–109°02′E) in Hainan, China; and on *B. fruticosa* and *B. rostrata* in Wanshi Botanical Garden (alt. 28–201 m, 117°53′–118°25′E, 24°25′–24°54′N) and Tianzhu Mountain National Forest Park (alt. 50–200 m, 24°35′–24°39′N, 117°53′–117°57′E) in Fujian, China.

### Studied Insects and Plants

*Epicephala lativalvaris* Li, Wang & Zhang, 2012 and *E. mirivalvata* Li, Wang & Zhang, 2012 are two small nocturnal moths that are distinctly different in morphology [17]. Females have a different oviposition mode due to having a differently shaped ovipositor (Figure 5). However, they obligately and jointly pollinated *B. fruticosa* in Hainan and Fujian, and pollinated *B. rostrata* in Fujian.

*Breynia fruticosa* and *B. rostrata* are similar in morphology: male flowers (Figure 6: A, B) have a funnel-shaped calyx with six dentations at apex; the three stamens fused into a column and concealed in calyx, which makes it hard to be touched by flower visitors except *Epicephala* moths; female flowers bear free calyx and styles, which is likely to be accessed by other flower visitors. The two *Breynia* species can be distinguished by having different calyx and stigmas: in *B. fruticosa* (Figure 6: C), calyx sepals radially spread into a disk, stigmas are excurved and calyx sepals are enlarged in fruit (Figure 6: D); in *B. rostrata* (Figure 6: E), calyx sepals are reflexed, stigmas are erect and calyx sepals are not enlarged in fruit (Figure 6: F).

### Methods

*Breynia fruticosa* flowered for the 10 warmer months of the year, fruited sparsely and irregularly in Hong Kong [18]. In order to acquire detailed phenological data of *B. fruticosa* and *B. rostrata* as to compare with the life history of *E. mirivalvata* and *E. lativalvaris*, we made a tracking observation of the flower and fruit developmental stages of *B. fruticosa* in Hainan, as well as of *B. fruticosa* and *B. rostrata* in Fujian. We randomly selected ten individuals of *B. fruticosa* in Yingge Mountain Nature Reserves, and five individuals of both *B. fruticosa* and *B. rostrata* in Tianzhu Mountain National Forest Park and Wanshi Botanical Garden, respectively. We recorded the development of flowers and fruits, and counted the number of flowers, the fruits and developing fruits of the selected plant individuals once a week.

To make sure whether the hybridization could happen between *B. fruticosa* and *B. rostrata*, we performed a hand-pollination hybridization experiment in Wanshi Botanical Garden. We respectively selected 50 non-pollinating female flowers of *B. fruticosa* and *B. rostrata*. We gathered pollen grains from male flowers of *B. fruticosa* and hand-pollinated female flowers of *B. rostrata*, and from male flowers of *B. rostrata* and hand-pollinated female flowers of *B. fruticosa*. We bagged these pollinated flowers with fine netting (0.5 mm mesh), and seven days later, we counted and calculated their developmental rate (Figure S2).

We observed *Epicephala* moths during full anthesis and recorded their visiting behavior in detail, with particular attention paid to their nocturnal activities. We recorded the time that the moths spent on pollination, oviposition and pollen collection, and observed how they used their proboscis to collect pollen and pollinate flowers, and where they oviposited. We collected developing and mature fruits and put them in a cylindrical plastic box (8.5 cm×12.0 cm) to rear *Epicephala* larvae. We then recorded how and when the larvae left fruits to cocoon and emerge.

The presence of oviposition scars and eggs on fruits was considered to have a one–to–one correspondence [11]. We randomly examined 23 flowers in Hainan (Yingge Mountain) and 179 flowers in Fujian (Tianzhu Mountain and Wanshi Botanical Garden) to search for eggs (Table S3); and randomly examined oviposition scars on 1162 fruits in Hainan (Yingge Mountain, Wuzhi Mountain and Jianfeng Mountain) and 974 fruits in Fujian (Tianzhu Mountain and Wanshi Botanical Garden) to acquire the egg amount of *Epicephala* moths (Table S4).

Adult specimens examined in this study were collected from their hosts or reared from fruits in captivity. Dissection of female flowers of *Breynia* plants and female genitalia of *Epicephala* moths was conducted under an Olympus SZ11 stereo-microscope. Photographs were taken with a Canon G10 digital camera in the field and the illustration of genitalia were prepared with an Olympus C–7070 Wide Zoom digital camera.

We extracted genomic DNA of *Epicephala* moths from the fruits we reared, and extracted genomic DNA of *B. rostrata* from silica-gel dried leaves collected in Tianzhu Mountain, Fujian. The method of DNA extraction followed the CTAB procedure [19]. We PCR-amplified the *COI* gene fragments using primers LepF1 and LepR1 [20], and PCR-amplified the *matK* gene fragments using primers 570F and 1710R [21].

We analyzed the phylogenetic relationship between *E. lativalvaris* and *E. mirivalvata* based on the mitochondrial cytochrome oxidase subunit I (*COI*) gene of 20 *Epicephala* sequences, which is widely used in the phylogenetic analysis of insects [9], [22], [23]. The sequences of *E. lativalvaris*, *E. mirivalvata* and *E. relictella* were newly obtained during this study and 17 sequences were adopted from the previous studies [8]–[9] (Table S5). Alignment of *COI* gene and *matK* gene were aligned using Mega v. 5.05. The phylogeny was rooted with *Epicephala* sp. ex *Flueggea suffruticosa* according to the phylogenetic analysis of *Epicephala* and Phyllantheae [10].

We analyzed the phylogenetic relationship between *B. fruticosa* and *B. rostrata* based on the maturase K (*matK*) gene, which has previously been shown to be particularly useful for phylogenetic analysis of the family and tribe [8], [9], [21], [24]. *Breynia rostrata* sequence was newly obtained during this study and the remaining sequences were adopted from the previous studies [8], [9], [21] (Table S6). The phylogeny was rooted with *F. suffruticosa* according to the phylogenetic analysis of *Epicephala* and Phyllantheae [10].

Maximum-parsimony and maximum-likelihood analyses of *COI* and *matK* were performed using PAUP v. 4.0 b10 [25]. Both maximum-parsimony and maximum-likelihood heuristic searches were conducted with equal weight, 1000 and 100 replicates of random addition analyses respectively, and tree bisection-reconnection branch swapping. The robustness of maximum-parsimony and maximum-likelihood trees were assessed by non-parametric bootstrapping with 1000 and 100 replicates respectively.

## Supporting Information

Figure S1 *Epicephala* moths sucking nectar on female flowers of *Breynia fruticosa* (A) and *B. rostrata* (B).
(TIF)

Figure S2 Hand-pollination hybridization experiment of *Breynia fruticosa* and *B. rostrata*. (A) flowers bagged with fine netting. (B) hand-pollinated *B. rostrata* with pollen of *B. fruticosa*. (C) non-pollinated female flower of *B. fruticosa*. (D) developed female flowers of *B. fruticosa* with pollen of *B. rostrata*. (E) non-pollinated female flower of *B. rostrata*. (F) developed female flowers of *B. rostrata* with pollen of *B. fruticosa*.
(TIF)

Table S1 Annual fruit stage of *Breynia fruticosa* and life history of *Epicephala lativalvaris* and *E. mirivalvata* in Yingge Mountain of Hainan, China.
(DOC)

Table S2 Annual fruit stage of *Breynia fruticosa* and *Epicephala rostrata*, and life history of *E. lativalvaris* and *E. mirivalvata* in Tianzhu Mountain and Wanshi Botanical Garden of Xiamen, China.
(DOC)

Table S3 Statistics of oviposition scars made by *Epicephala lativalvaris* on *Breynia* fruits at five locations.
(DOC)

Table S4 Statistics of eggs laid by *Epicephala mirivalvata* on *Breynia* female flowers at three locations.
(DOC)

Table S5 GenBank accession number of *Epicephala* moths *COI* sequences.
(DOC)

Table S6 GenBank accession number of Phyllantheae plants *matK* sequences.
(DOC)

Table S7 Developed set of *Breynia* female flowers in hand-pollination experiment.
(DOC)
