# Supplementary figures and images for: Diffuse Coevolution between Two Epicephala Species (Gracillariidae) and Two Breynia Species (Phyllanthaceae)
Source: PLoS One. 2012 Jul 27;7(7):e41657. doi: 10.1371/journal.pone.0041657 (PMC3407192; doi:10.1371/journal.pone.0041657)

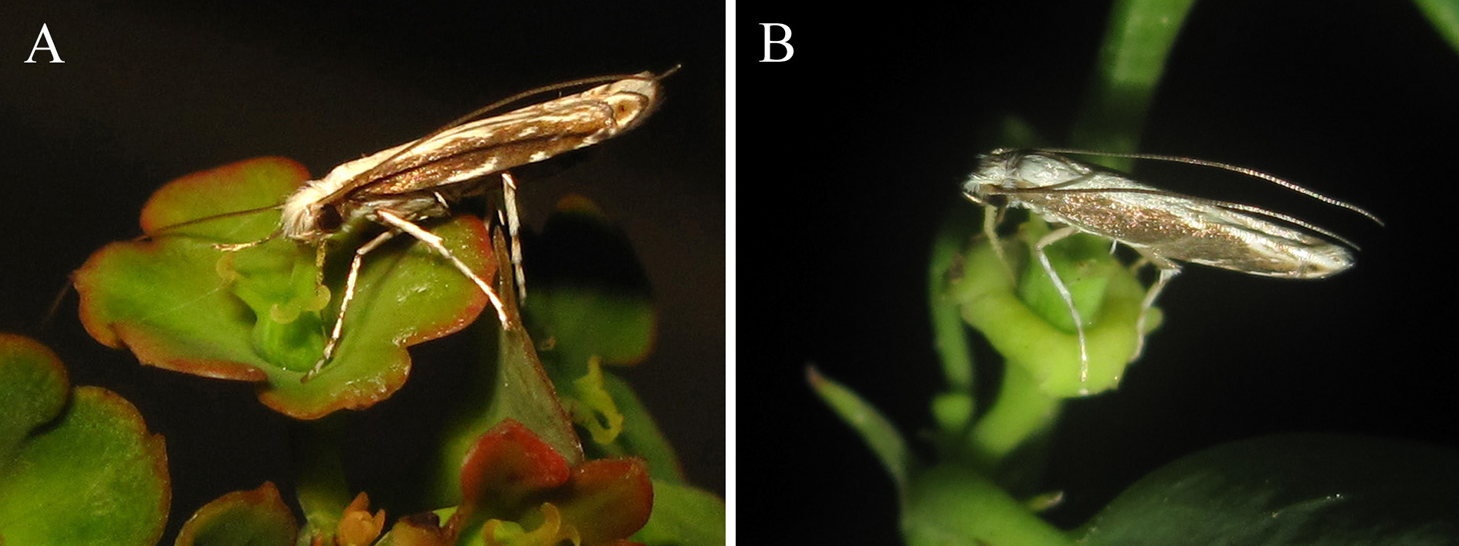

Supplement: Figure S1 — Epicephala moths sucking nectar on female flowers of Breynia fruticosa (A) and B. rostrata (B). (TIF) [file pone.0041657.s001.tif]

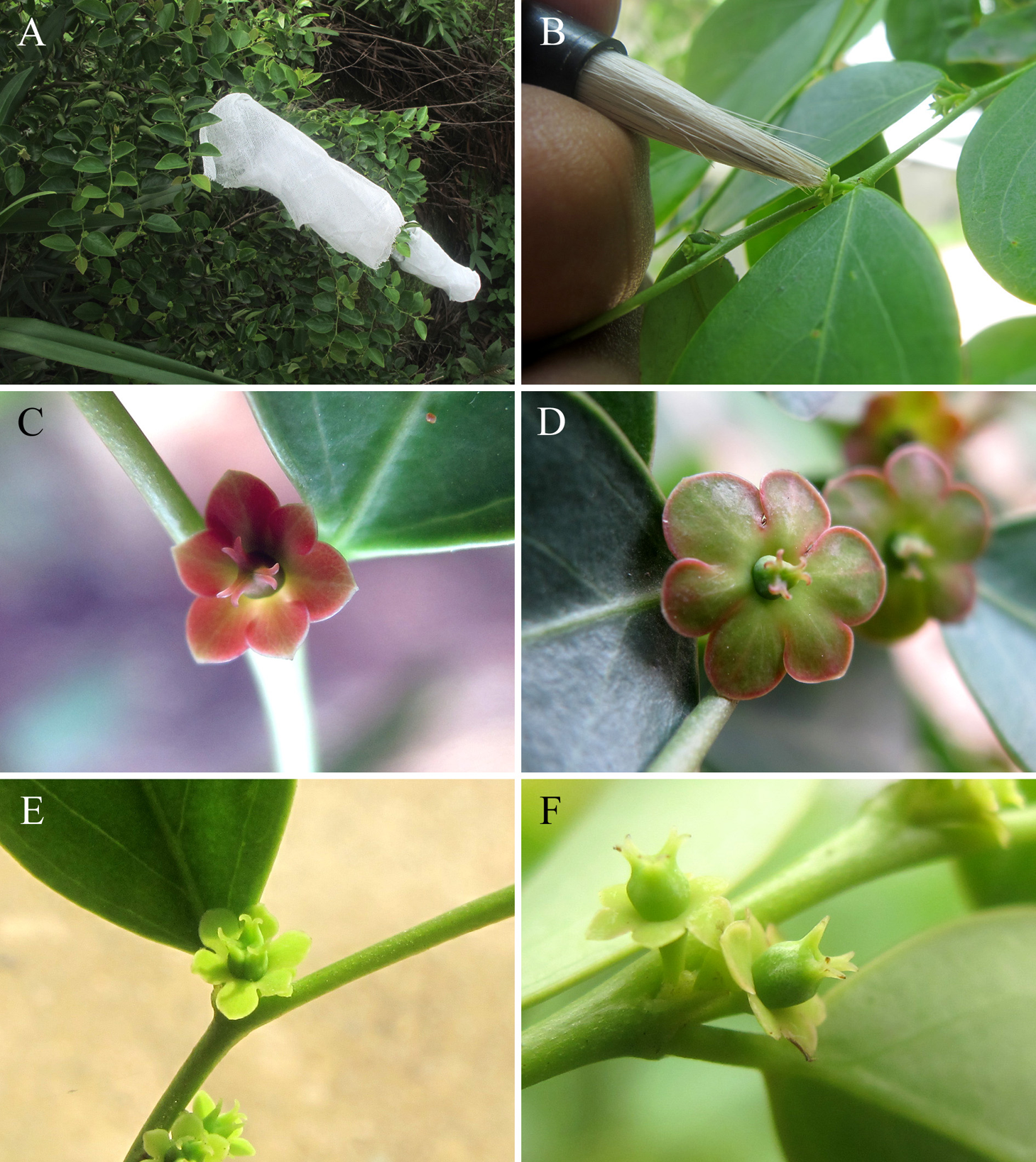

Supplement: Figure S2 — Hand-pollination hybridization experiment of Breynia fruticosa and B. rostrata. (A) flowers bagged with fine netting. (B) hand-pollinated B. rostrata with pollen of B. fruticosa. (C) non-pollinated female flower of B. fruticosa. (D) developed female flowers of B. fruticosa with pollen of B. rostrata. (E) non-pollinated female flower of B. rostrata. (F) developed female flowers of B. rostrata with pollen of B. fruticosa. (TIF) [file pone.0041657.s002.tif]
