# Supplementary material for: Diffuse Coevolution between Two Epicephala Species (Gracillariidae) and Two Breynia Species (Phyllanthaceae)
Source: PLoS One. 2012 Jul 27;7(7):e41657. doi: 10.1371/journal.pone.0041657 (PMC3407192; doi:10.1371/journal.pone.0041657)
Supplement: Table S2 — Annual fruit stage of Breynia fruticosa and Epicephala rostrata, and life history of E. lativalvaris and E. mirivalvata in Tianzhu Mountain and Wanshi Botanical Garden of Xiamen, China. (DOC) [file pone.0041657.s004.doc]

**Table S2.** Annual fruit stage of *Breynia fruticosa* and *Epicephala rostrata*, and life history of *E. lativalvaris* and *E. mirivalvata* in Tianzhu Mountain and Wanshi Botanical Garden of Xiamen, China.

| Generation | Mar | | | Apr | | | May | | | Jun | | | Jul | | | Aug | | | Sep | | | Oct | | | Nov | | | Dec–Feb | | |
| --- | --- | --- | --- | --- | --- | --- | --- | --- | --- | --- | --- | --- | --- | --- | --- | --- | --- | --- | --- | --- | --- | --- | --- | --- | --- | --- | --- | --- | --- | --- |
|  | F | M | L | F | M | L | F | M | L | F | M | L | F | M | L | F | M | L | F | M | L | F | M | L | F | M | L | F | M | L |
| 1st |  |  |  |  |  |  |  |  |  | ■ | ■ |  |  |  |  |  |  |  |  |  |  |  |  |  |  |  |  |  |  |  |
|  |  |  |  |  |  | ● | ● | ● |  |  |  |  |  |  |  |  |  |  |  |  |  |  |  |  |  |  |  |  |  |  |
|  |  |  |  |  |  |  | − | − | − | − |  |  |  |  |  |  |  |  |  |  |  |  |  |  |  |  |  |  |  |  |
|  |  |  |  |  |  |  |  |  |  | □ | □ | □ | □ |  |  |  |  |  |  |  |  |  |  |  |  |  |  |  |  |  |
|  |  |  |  |  |  |  |  |  |  |  | + | + | + |  |  |  |  |  |  |  |  |  |  |  |  |  |  |  |  |  |
| 2nd |  |  |  |  |  |  |  |  |  |  |  |  |  |  | ■ | ■ |  |  |  |  |  |  |  |  |  |  |  |  |  |  |
|  |  |  |  |  |  |  |  |  |  |  | ● | ● | ● |  |  |  |  |  |  |  |  |  |  |  |  |  |  |  |  |  |
|  |  |  |  |  |  |  |  |  |  |  |  | − | − | − | − |  |  |  |  |  |  |  |  |  |  |  |  |  |  |  |
|  |  |  |  |  |  |  |  |  |  |  |  |  |  |  | □ | □ | □ | □ |  |  |  |  |  |  |  |  |  |  |  |  |
|  |  |  |  |  |  |  |  |  |  |  |  |  |  |  |  | + | + | + |  |  |  |  |  |  |  |  |  |  |  |  |
| 3rd |  |  |  |  |  |  |  |  |  |  |  |  |  |  |  |  |  |  |  | ■ | ■ | ■ |  |  |  |  |  |  |  |  |
|  |  |  |  |  |  |  |  |  |  |  |  |  |  |  |  | ● | ● | ● |  |  |  |  |  |  |  |  |  |  |  |  |
|  |  |  |  |  |  |  |  |  |  |  |  |  |  |  |  |  | − | − | − | − | − | − |  |  |  |  |  |  |  |  |
|  |  |  |  |  |  |  |  |  |  |  |  |  |  |  |  |  |  |  |  | □ | □ | □ | □ |  |  |  |  |  |  |  |
|  |  |  |  |  |  |  |  |  |  |  |  |  |  |  |  |  |  |  |  |  | + | + | + |  |  |  |  |  |  |  |
| 4th |  |  |  |  |  |  |  |  |  |  |  |  |  |  |  |  |  |  |  |  |  |  |  |  | ■ | ■ |  |  |  |  |
|  |  |  |  |  |  |  |  |  |  |  |  |  |  |  |  |  |  |  |  |  | ● | ● | ● |  |  |  |  |  |  |  |
|  |  |  |  |  |  |  |  |  |  |  |  |  |  |  |  |  |  |  |  |  |  | − | − | − | − | − | − |  |  |  |
|  | (□) | (□) | (□) | (□) | (□) | (□) | (□) | (□) |  |  |  |  |  |  |  |  |  |  |  |  |  |  |  |  | □ | □ | □ | (□) | (□) | (□) |
|  |  |  |  |  |  | + | + | + |  |  |  |  |  |  |  |  |  |  |  |  |  |  |  |  |  | + | + |  |  |  |
| 5th |  |  |  |  | ■ | ■ |  |  |  |  |  |  |  |  |  |  |  |  |  |  |  |  |  |  |  |  |  |  |  |  |
|  | (●) | (●) |  |  |  |  |  |  |  |  |  |  |  |  |  |  |  |  |  |  |  |  |  |  |  | (●) | (●) | (●) | (●) | (●) |
|  |  | − | − | − | − | − | − |  |  |  |  |  |  |  |  |  |  |  |  |  |  |  |  |  |  |  |  |  |  |  |
|  |  |  |  |  |  | □ | □ | □ |  |  |  |  |  |  |  |  |  |  |  |  |  |  |  |  |  |  |  |  |  |  |
|  |  |  |  |  |  |  |  | + |  |  |  |  |  |  |  |  |  |  |  |  |  |  |  |  |  |  |  |  |  |  |

■ Fruit stage. ● Egg, − Larva. □ Pupa. + Adult. F: First ten days. M: Middle ten days. L: Last ten days.
