# Supplementary material for: Diffuse Coevolution between Two Epicephala Species (Gracillariidae) and Two Breynia Species (Phyllanthaceae)
Source: PLoS One. 2012 Jul 27;7(7):e41657. doi: 10.1371/journal.pone.0041657 (PMC3407192; doi:10.1371/journal.pone.0041657)
Supplement: Table S3 — Statistics of oviposition scars made by Epicephala lativalvaris on Breynia fruits at five locations. (DOC) [file pone.0041657.s005.doc]

**Table S3.** Statistics of oviposition scars made by *Epicephala lativalvaris* on *Breynia* fruits at five locations.

| Locality | Host | Fruits examined | Intact fruits | Fruits with oviposition scars | Intact fruits with oviposition scars | Proportion of fruits with ovipositon scars ( %) |
| --- | --- | --- | --- | --- | --- | --- |
| YGL | *B. fruticosa* | 1049 | 713 | 753 | 426 | 71.78 |
| JFL | *B. fruticosa* | 76 | 59 | 48 | 31 | 63.16 |
| WZS | *B. fruticosa* | 37 | 24 | 24 | 14 | 64.86 |
| TZS | *B. fruticosa* | 63 | 41 | 51 | 29 | 80.95 |
| WS | *B. fruticosa* | 44 | 15 | 38 | 9 | 86.36 |
| sum | *B. fruticosa* | 1269 | 852 | 914 | 502 | 72.03 |
| TZS | *B. rostrata* | 108 | 22 | 95 | 26 | 87.96 |
| WS | *B. rostrata* | 759 | 281 | 673 | 195 | 88.67 |
| sum | *B. rostrata* | 867 | 303 | 768 | 221 | 88.58 |

YGL: Yingge Mountain, Hainan. JFL: Jianfeng Mountain, Hainan. WZS: Wuzhi Mountain, Hainan. TZS: Tianzhu Mountain, Fujian. WS: Wanshi Botanical Garden, Fujian.
