# Supplementary material for: Diffuse Coevolution between Two Epicephala Species (Gracillariidae) and Two Breynia Species (Phyllanthaceae)
Source: PLoS One. 2012 Jul 27;7(7):e41657. doi: 10.1371/journal.pone.0041657 (PMC3407192; doi:10.1371/journal.pone.0041657)
Supplement: Table S4 — Statistics of eggs laid by Epicephala mirivalvata on Breynia female flowers at three locations. (DOC) [file pone.0041657.s006.doc]

**Table S4.** Statistics of eggs laid by *Epicephala mirivalvata* on *Breynia* female flowers at three locations

| Locality | Host | Female flowers examined | Female flowers with egg | Proportion of female flowers with egg (%) |
| --- | --- | --- | --- | --- |
| YGL | *B. fruticosa* | 23 | 1 | 4.35 |
| WS | *B. fruticosa* | 8 | 0 | 0 |
| TZS | *B. fruticosa* | 45 | 1 | 2.22 |
| sum | *B. fruticosa* | 76 | 2 | 2.63 |
| WS | *B. rostrata* | 51 | 3 | 5.88 |
| TZS | *B. rostrata* | 75 | 2 | 2.67 |
| sum | *B. rostrata* | 126 | 5 | 3.97 |

YGL: Yingge Mountain, Hainan. TZS: Tianzhu Mountain, Fujian. WS: Wanshi Botanical Garden, Fujian.
