# Supplementary material for: Diffuse Coevolution between Two Epicephala Species (Gracillariidae) and Two Breynia Species (Phyllanthaceae)
Source: PLoS One. 2012 Jul 27;7(7):e41657. doi: 10.1371/journal.pone.0041657 (PMC3407192; doi:10.1371/journal.pone.0041657)
Supplement: Table S5 — GenBank accession number of Epicephala moths COI sequences. (DOC) [file pone.0041657.s007.doc]

**Table S5.** GenBank accession number of *Epicephala* moths *COI* sequences

| Number | GenBank accession numbers | *Epicephala* species related with host |
| --- | --- | --- |
| 1 | *E.* sp. ex *Breynia oblongifolia* | FJ235381 |
| 2 | *E.* sp. ex *Breynia vitis-idaea* | FJ235380 |
| 3 | *E.* sp. ex *Breynia fruticosa* | FJ235379 |
| 4 | *E.* sp. ex *Breynia disticha* | FJ235378 |
| 5 | *E. mirivalvaris* | JX231168 |
| 6 | *E. reticulatus* | JF797232 |
| 7 | *E. lativalvaris* | JX231167 |
| 8 | *E.* sp. ex *Phyllanthus amarus* | FJ235388 |
| 9 | *E.* sp. ex *Phyllanthus ussuriensis* | FJ235387 |
| 10 | *E.* sp. ex *Phyllanthus lepidocarpus* | FJ235386 |
| 11 | *E.* sp. ex *Phyllanthus reticulatus* | FJ235383 |
| 12 | *E.* sp. ex *Phyllanthus vulcani* | FJ235377 |
| 13 | *E.* sp. ex *Phyllanthus caudatus* | FJ235375 |
| 14 | *E.* sp. ex *Phyllanthus koniamboensis* | FJ235374 |
| 15 | *E.* sp. ex *Flueggea suffruticosa* | FJ235373 |
| 16 | *E.* sp. ex *Glochidion zeylanicum* | DQ299039 |
| 17 | *E.* sp. ex *Glochidion obovatum*/*Glochidion rubrum* | DQ299027 |
| 18 | *E.* sp. ex *Glochidion acuminatum* | DQ298955 |
| 19 | *E.* sp. ex *Glochidion lanceolatum* | DQ298967 |
| 20 | *E.* sp. ex *Glochidion obovatum*/*Glochidion rubrum* | DQ299009 |
