# Supplementary material for: Diffuse Coevolution between Two Epicephala Species (Gracillariidae) and Two Breynia Species (Phyllanthaceae)
Source: PLoS One. 2012 Jul 27;7(7):e41657. doi: 10.1371/journal.pone.0041657 (PMC3407192; doi:10.1371/journal.pone.0041657)
Supplement: Table S6 — GenBank accession number of Phyllantheae plants matK sequences. (DOC) [file pone.0041657.s008.doc]

**Table S6.** GenBank accession number of Phyllantheae plants *matK* sequences

| Number | Hostplants of *Epicephala* species | GenBank accession numbers |
| --- | --- | --- |
| 1 | *Flueggea suffruticosa* | AY552427 |
| 2 | *Breynia stipitata* | AY552422 |
| 3 | *Breynia cernua* | AY552423 |
| 4 | *Breynia disticha* | AY936564 |
| 5 | *Breynia rostrata* | JX231166 |
| 6 | *Breynia fruticosa* | FJ235245 |
| 7 | *Breynia oblongifolia* | FJ235242 |
| 8 | *Breynia retusa* | AY936565 |
| 9 | *Breynia* *vitis-idaea* | AY936566 |
| 10 | *Glochidion acuminatum* | FJ235239 |
| 11 | *Glochidion lanceolatum* | FJ235235 |
| 12 | *Glochidion obovatum* | FJ235238 |
| 13 | *Glochidion rubrum* | FJ235236 |
| 14 | *Glochidion zeylanicum* | FJ235237 |
| 15 | *Phyllanthus aeneus* | FJ235260 |
| 16 | *Phyllanthus amarus* | AY936574 |
| 17 | *Phyllanthus bourgeoisii* | FJ235256 |
| 18 | *Phyllanthus chamaecerasus* | AY936583 |
| 19 | *Phyllanthus guillauminii* | FJ235261 |
| 20 | *Phyllanthus koniamboensis* | FJ235258 |
| 21 | *Phyllanthus mangenotii* | FJ235257 |
| 22 | *Phyllanthus reticulatus* | AY936629 |
