# Supplementary material for: Diffuse Coevolution between Two Epicephala Species (Gracillariidae) and Two Breynia Species (Phyllanthaceae)
Source: PLoS One. 2012 Jul 27;7(7):e41657. doi: 10.1371/journal.pone.0041657 (PMC3407192; doi:10.1371/journal.pone.0041657)
Supplement: Table S7 — Developed set of Breynia female flowers in hand-pollination experiment. (DOC) [file pone.0041657.s009.doc]

**Table S7.** Developped set of *Breynia* female flowers in hand-pollination experiment

|  | Number of female flowers | Number of developed female flowers | Flower developed set |
| --- | --- | --- | --- |
| *B. fruticosa* individuals | 50 | 45 | 90% |
| *B. rostrata* individuals | 50 | 47 | 94% |
